# Primary cervical screening with high risk human papillomavirus testing: observational study

**DOI:** 10.1136/bmj.l240

**Published:** 2019-02-06

**Authors:** Matejka Rebolj, Janet Rimmer, Karin Denton, John Tidy, Christopher Mathews, Kay Ellis, John Smith, Chris Evans, Thomas Giles, Viki Frew, Xenia Tyler, Alexandra Sargent, Janet Parker, Miles Holbrook, Katherine Hunt, Penny Tidbury, Tanya Levine, David Smith, Julietta Patnick, Ruth Stubbs, Sue Moss, Henry Kitchener

**Affiliations:** 1 Centre for Cancer Prevention, Wolfson Institute of Preventive Medicine, Barts & The London School of Medicine and Dentistry, Queen Mary University of London, London, UK; 2 School of Cancer and Pharmaceutical Sciences, Faculty of Life Sciences and Medicine, King’s College London, London SE1 9RT, UK; 3 Young Person and Adult Screening Programmes, Public Health England, Sheffield, UK; 4 PHE Screening Quality Assurance Service South, Public Health England, Bristol, UK; 5 Severn Pathology, Southmead Hospital, North Bristol NHS Trust, Bristol, UK; 6 Department of Gynaecological Oncology, Royal Hallamshire Hospital, Sheffield Teaching Hospitals NHS Foundation Trust, Sheffield, UK; 7 Cytology, Royal Hallamshire Hospital, Sheffield Teaching Hospitals NHS Foundation Trust, Sheffield, UK; 8 NHS Liverpool Clinical Laboratories, Royal Liverpool University Hospital, Liverpool, UK; 9 Department of Cellular Pathology, Norfolk & Norwich University Hospitals NHS Foundation Trust, Norwich, UK; 10 Clinical Virology, Manchester University NHS Foundation Trust, Manchester, UK; 11 Cellular Pathology, Manchester University NHS Foundation Trust, Manchester, UK; 12 Department of Cellular Pathology, Northwick Park Hospital, London, UK; 13 Cancer Epidemiology Unit, Nuffield Department of Population Health, University of Oxford, Oxford, UK; 14 Division of Cancer Sciences, University of Manchester and Manchester NIHR BRC, Manchester, UK

## Abstract

**Objective:**

To provide the first report on the main outcomes from the prevalence and incidence rounds of a large pilot of routine primary high risk human papillomavirus (hrHPV) testing in England, compared with contemporaneous primary liquid based cytology screening.

**Design:**

Observational study.

**Setting:**

The English Cervical Screening Programme.

**Participants:**

578 547 women undergoing cervical screening in primary care between May 2013 and December 2014, with follow-up until May 2017; 183 970 (32%) were screened with hrHPV testing.

**Interventions:**

Routine cervical screening with hrHPV testing with liquid based cytology triage and two early recalls for women who were hrHPV positive and cytology negative, following the national screening age and interval recommendations.

**Main outcome measures:**

Frequency of referral for a colposcopy; adherence to early recall; and relative detection of cervical intraepithelial neoplasia grade 2 or worse from hrHPV testing compared with liquid based cytology in two consecutive screening rounds.

**Results:**

Baseline hrHPV testing and early recall required approximately 80% more colposcopies, (adjusted odds ratio 1.77, 95% confidence interval 1.73 to 1.82), but detected substantially more cervical intraepithelial neoplasia than liquid based cytology (1.49 for cervical intraepithelial neoplasia grade 2 or worse, 1.43 to 1.55; 1.44 for cervical intraepithelial neoplasia grade 3 or worse, 1.36 to 1.51) and for cervical cancer (1.27, 0.99 to 1.63). Attendance at early recall and colposcopy referral were 80% and 95%, respectively. At the incidence screen, the 33 506 women screened with hrHPV testing had substantially less cervical intraepithelial neoplasia grade 3 or worse than the 77 017 women screened with liquid based cytology (0.14, 0.09 to 0.23).

**Conclusions:**

In England, routine primary hrHPV screening increased the detection of cervical intraepithelial neoplasia grade 3 or worse and cervical cancer by approximately 40% and 30%, respectively, compared with liquid based cytology. The very low incidence of cervical intraepithelial neoplasia grade 3 or worse after three years supports extending the screening interval.

## Introduction

Randomised trials have shown that cervical screening for high risk human papillomavirus (hrHPV) achieves greater sensitivity than cytology in the detection of cervical intraepithelial neoplasia and greater protection against cervical cancer.^1^ ^2^ ^3^ ^4^ ^5^ Because of the enhanced sensitivity, screening intervals can be safely extended.^5^ ^6^ However, hrHPV testing has reduced specificity compared with cytology, due to the high prevalence of hrHPV.^7^ Excessive referral for a colposcopy can be mitigated by triage of positive hrHPV test results by using reflex liquid based cytology. Most women who are hrHPV positive who undergo reflex cytology will have a normal result (cytology negative), however, this group of women remain at increased risk either because of underlying disease not detected by cytology, or because of an increased risk of incident disease.^5^ ^8^ Alternative strategies for management for this group are either referral for a colposcopy for women who are at greatest risk of underlying disease by virtue of being human papillomavirus genotype 16/18 positive, or to defer a colposcopy anticipating evidence of clearance of the hrHPV infection (which will occur in around 40% by 12 months),^9^ thus allowing these women to be returned safely to routine recall.

The UK National Screening Committee recommended a switch to hrHPV primary screening in January 2016. NHS England and Public Health England are now working towards a national roll-out by the end of 2019. In support of the anticipated roll-out, a pilot was established in 2013 involving a group of screening laboratories in the NHS Cervical Screening Programme in England.^10^ ^11^ Conversion to primary hrHPV was partial, which allowed for comparison with contemporaneous primary liquid based cytology. This recognised the importance of implementing primary hrHPV screening in a controlled manner, enabling evaluation of a protocol with respect to practicability, acceptability, and cost effectiveness. The value of the pilot in terms of learning was further enhanced by a prespecified variation in protocol, ahead of a national roll-out.

Here, we studied baseline referral for a colposcopy and the extent of additional colposcopy referrals as a result of early recall of women who were hrHPV positive and cytology negative; adherence to early recall; the sensitivity of hrHPV testing in the prevalence round and the incidence of high grade cervical intraepithelial neoplasia among women who were hrHPV negative at three years.

## Methods

Six NHS laboratories had partially converted to primary hrHPV testing between May and August 2013. These laboratories in Bristol, Liverpool, Manchester, Norwich, Northwick Park (West London), and Sheffield represent approximately 13% of the Cervical Screening Programme. Conversion, which involved around one third of screening samples, was population based. Distribution of primary hrHPV testing was based on clusters of general practices and was not subject to random allocation. Rather, allocation to liquid based cytology or hrHPV testing was a consequence of practical considerations such as maintaining one management protocol for each colposcopy unit, whereby colposcopy units often serve defined administrative clusters. Samples from women who were screened after a routine invitation were collected in primary care in either ThinPrep (Hologic, Marlborough, MA) or SurePath (Beckton Dickinson, Sparks, MD) liquid based cytology media, regardless of whether liquid based cytology or hrHPV was the primary screening test. hrHPV testing was performed with either the Cobas 4800 (Roche, Rotkreuz, Switzerland, or Branchburg, NJ), RealTime (Abbott, Wiesbaden, Germany), APTIMA (Hologic, Manchester, UK), or, to a limited degree, Hybrid Capture 2 (Qiagen, Gaithersburg, MD) assays. Each laboratory used a unique combination of liquid based cytology medium and hrHPV assay. All screening tests have been approved for use in the English Cervical Screening Programme following the official validation protocols.

The pilot adhered to the nationally recommended age range (25-64) and screening intervals (three years for women aged <50 and five years thereafter) for both screening tests. In women screened with hrHPV testing, cytology was not blinded to the hrHPV test result. Women were immediately referred for a colposcopy if their hrHPV test was positive and cytology showed any grade of abnormality. Women who were hrHPV positive with negative cytology were recommended for early recall at 12 months, at which point they were referred for a colposcopy if they remained persistently hrHPV positive and had developed any cytological abnormalities. Three laboratories referred women with persistently negative cytology at 12 months if their samples showed persistent infection with HPV 16/18. Other women who were hrHPV positive and cytology negative were offered further early recall at 24 months, and were referred for a colposcopy if they showed a persistent hrHPV infection regardless of their cytology. Women screened with cytology were recommended for a colposcopy if their cytology showed high grade abnormalities consistent with high grade squamous intraepithelial lesions in Bethesda 2001 terminology or low grade abnormalities consistent with either atypical squamous cells of undetermined significance (ASCUS) or low grade squamous intraepithelial lesions combined with a positive reflex test for hrHPV. Cytological glandular abnormalities were classified among high grade squamous intraepithelial lesions. Women not referred to colposcopy or early recall were routinely recalled at three or five years.

Screening was conducted in primary care, and training in relation to hrHPV based screening was provided. The laboratories routinely monitored compliance with referral for a colposcopy, and there was a safety net in place for women who did not attend. Colposcopy was conducted according to national clinical practice guidelines. All laboratories and colposcopy clinics took part in the national quality assurance programmes.

### Data sources and linkage

We retrieved records for screening samples taken by general practitioners and information on the associated referrals for a colposcopy from the pilot laboratories’ information systems. The data were collated, processed, and analysed centrally at Queen Mary University of London. For the present report, complete data were available until 31 May 2017. We linked tests from the same woman by using unique English NHS numbers. For the period starting two years preceding the pilot, the date of sample and recommendation were collected. We excluded index samples that had been preceded by another within the prior two years, because they were probably not taken for primary screening. We also excluded index samples if their management code identified the sample to have been taken as follow-up or at the time of colposcopy. Episodes started with the first (index) screening test and were closed depending on this test’s outcome and any subsequent tests (supplementary material). If an initial inadequate test (0.3% with hrHPV testing, and 2.3% with liquid based cytology) was followed by a valid test, we used the result of the latter for analyses. The first episode for each woman was considered the prevalence episode with respect to the initiation of the pilot; subsequent episodes were incidence episodes.

### Statistical analysis

We included all women aged 24-64 because the initial screening invitation is sent six months before a woman turns 25 years. We based the main analyses on prevalence episodes that had started by 31 December 2014 to provide complete cumulative data including early recall outcomes. These women had 29 months or more of follow-up in the available data. A subset of these women, those with screening samples processed at three laboratories that used human papillomavirus genotyping for management of women at the 12 month early recall, provided human papillomavirus genotyping data. We included incidence episodes if women aged 24-46 at the prevalence episode had been directed to routine recall in three years.

We tested differences in the distribution of sample characteristics at the index test of the prevalence round with χ^2^. We defined a positive screening test result as a result with a known screening and triage test result that required further clinical management according to the recommended protocol. We calculated odds ratios and their 95% confidence intervals for differences between hrHPV and cytology screening with logistic regression, and adjusted for the woman’s age, processing laboratory, and decile of index of multiple deprivation. The robustness of the results for the two main outcomes, the number of colposcopies and cervical intraepithelial neoplasia grade 2 or worse, was additionally studied by applying a Mantel-Haenszel test to 240 strata defined by laboratory (six sites), index of multiple deprivation (five quintiles) and five year age group (eight groups: 24-29, 30-34, 35-39, 40-44, 45-49, 50-54, 55-59, and 60-64). We based the index of multiple deprivation on the woman’s postcode at the time of her index sample (supplementary material). We excluded 9689 (1.6%) from the total of 578 547 women entering the pilot until 31 December 2014 for having an unknown postcode.

All data management and statistical analyses were undertaken with R version 3.2.4 and Oracle SQL Developer version 4.0.2.

### Patient and public involvement

Neither patients nor the public were involved in the management of this study. A parallel investigation on the psychological consequences of hrHPV testing compared with liquid based cytology screening embedded into the pilot will report its findings separately.^12^

## Results

### Baseline (prevalence) screening round and compliance with early recall

This on-going pilot included 1 532 908 women screened in the prevalence round until 31 May 2017 (supplementary table 1). The distribution by age was: 297 843 (19%) were aged 24-29, 850 088 (55%) 30-49, and 384 977 (25%) 50-64. Of these, 442 174 (29%) women were screened with hrHPV testing and 1 090 734 (71%) were screened with liquid based cytology. Overall recruitment between laboratories varied from 139 211 to 544 865, and 875 641 (57%) were from the most deprived areas (deciles 1-5 of the index of multiple deprivation). Table 1 shows that by 31 December 2014, 578 547 women had been screened, with a similar age and deprivation distribution as that across the entire pilot. Women screened with hrHPV testing were more likely to be from more affluent areas, and were marginally, but statistically significantly, older (χ^2^<0.0001). Additionally, the proportion of conversion to hrHPV screening differed by laboratory (χ^2^<0.0001).

**Table 1:** Comparison of populations and outcomes for high risk human papillomavirus (hrHPV) testing versus liquid based cytology (LBC) in the baseline (prevalence) screening round. Values are numbers (percentages) unless stated otherwise

Characteristic	hrHPV	LBC		Odds ratio for hrHPV testing ***v*** LBC (95% CI)	
				Unadjusted	Adjusted^*^
Total	183 970 (100)	394 577 (100)		NA	NA
Age at screening (years):					
24-29	35 085 (19)	75 847 (19)		NA	NA
30-49	105 365 (57)	226 034 (57)		NA	NA
50-64	43 520 (24)	92 696 (23)		NA	NA
IMD deciles at screening:					
1-5 (most deprived)	93 001 (51)	229 576 (58)		NA	NA
6-10 (least deprived)	90 969 (49)	165 001 (42)		NA	NA
Procedures (% screened)^†^:					
Positive screening test outcomes requiring additional testing	23 331 (12.7)^‡^	15 121 (3.8)^§^		3.64 (3.57 to 3.72)	3.90 (3.81 to 3.98)
Immediate referrals^¶^	7724 (4.2)	15 117 (3.8)		1.10 (1.07 to 1.13)	1.14 (1.11 to 1.17)
Referrals after repeated testing^¶^	5070 (2.8)	NR		NR	NR
Total referral^**^	13 010 (7.1)	18 205 (4.6)		1.57 (1.54 to 1.61)	1.61 (1.57 to 1.65)
Total colposcopies^**^	12 559 (6.8)	16 378 (4.2)		1.69 (1.65 to 1.73)	1.77 (1.73 to 1.82)
Histological outcomes, after immediate referral (% screened)^†¶^:					
CIN2+	3060 (1.7)	5932 (1.5)		1.11 (1.06 to 1.16)	1.12 (1.07 to 1.17)
CIN3+	1939 (1.1)	3753 (1.0)		1.11 (1.05 to 1.17)	1.12 (1.06 to 1.19)
Cervical cancer	89 (0.1)	167 (0.0)		1.14 (0.88 to 1.48)	1.14 (0.88 to 1.48)
Histological outcomes including immediate referral and early recall (% screened)^† ††:^					
Normal biopsy	6284 (3.4)	7126 (1.8)		1.92 (1.86 to 1.99)	1.98 (1.91 to 2.05)
CIN1	2039 (1.1)	2780 (0.7)		1.58 (1.49 to 1.67)	1.71 (1.62 to 1.82)
CIN2+	4156 (2.3)	6113 (1.6)		1.47 (1.41 to 1.53)	1.49 (1.43 to 1.55)
CIN3+	2521 (1.4)	3833 (1.0)		1.42 (1.35 to 1.49)	1.44 (1.36 to 1.51)
Cervical cancer	101 (0.1)	170 (0.0)		1.27 (1.00 to 1.63)	1.27 (0.99 to 1.63)

NA=not applicable; IMD=index of multiple deprivation; NR=not relevant; CIN2+=cervical intraepithelial neoplasia grade 2 or worse; CIN3+=cervical intraepithelial neoplasia grade 3 or worse; CIN1=cervical intraepithelial neoplasia grade 1.

* Adjusted for age (years), IMD decile, and laboratory.

† See supplementary figure 1A for hrHPV and 1B for LBC.

‡ hrHPV positive with a known cytological outcome.

§ hrHPV positive low grade abnormal cytology or high grade abnormal cytology regardless of the hrHPV status.

¶ Per protocol, in women with a record of referral to the specific type of follow-up (see supplementary figure 1A).

** Counted as one per woman, including referrals or colposcopies conforming to the screening recommendations and colposcopies in women with screening test results for which the screening recommendations did not include a referral for colposcopy.

†† Includes biopsies taken per protocol (colposcopy after immediate referral or after early recall at 12 and 24 months), and biopsies taken outside of the protocol (see supplementary figure 1).

**Fig 1:**
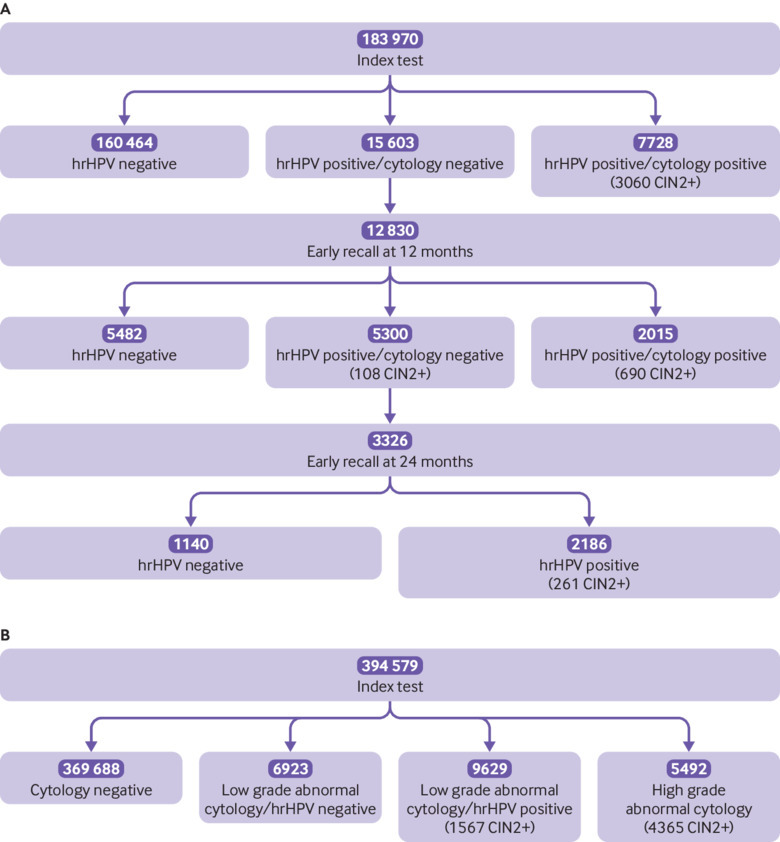
Flow diagram for prevalence episodes that started by 31 December 2014 including outcomes from per protocol follow-up until 31 May 2017 for women screened with high risk human papillomavirus (hrHPV) testing (A) and liquid based cytology (LBC) (B). CIN2+=cervical intraepithelial neoplasia grade 2 or worse

Figures 1A and 1B show, respectively, the per protocol detection of cervical intraepithelial neoplasia down the protocol pathway, for primary hrHPV and primary cytology (detailed outcomes including those outside of the protocol are reported in supplementary fig 1 and 2). Table 1 shows that hrHPV testing was positive in 12.7% of all screened women; 28.0% below age 30, 10.5% aged 30-49, and 5.6% aged 50-64 (table 2). Table 3 shows that about one third of hrHPV positive women were HPV 16/18 positive.

**Table 2:** Screening outcomes in the baseline (prevalence) screening round for high risk human papillomavirus (hrHPV) testing versus liquid based cytology (LBC), by age group

Outcome	Age group (years)				Total
	24-29	30-49	50-64		
**hrHPV (%)**					
Number	35 085 (100)	105 365 (100)	43 520 (100)		183 970 (100)
Positive screening test	9836 (28.0)	11 047 (10.5)	2448 (5.6)		23 331 (12.7)
Immediate referral for colposcopy	3795 (10.8)	3340 (3.2)	589 (1.4)		7724 (4.2)
Colposcopies	5826 (16.6)	5561 (5.3)	1172 (2.7)		12 559 (6.8)
CIN2+	2299 (6.6)	1638 (1.6)	219 (0.5)		4156 (2.3)
CIN3+	1403 (4.0)	1000 (0.9)	118 (0.3)		2521 (1.4)
**LBC (%)**					
Number	75 847 (100)	226 034 (100)	92 696 (100)		394 577 (100)
Positive screening test	7309 (9.6)	6678 (3.0)	1134 (1.2)		15 121 (3.8)
Immediate referral for colposcopy	7309 (9.6)	6675 (3.0)	1133 (1.2)		15 117 (3.8)
Colposcopies	7606 (10.0)	7436 (3.3)	1336 (1.4)		16 378 (4.2)
CIN2+	3319 (4.4)	2497 (1.1)	297 (0.3)		6113 (1.5)
CIN3+	2103 (2.8)	1555 (0.7)	175 (0.2)		3833 (1.0)
**Unadjusted odds ratio for hrHPV *v *LBC (95% CI)**					
Positive screening test	3.65 (3.53 to 3.78)	3.85 (3.73 to 3.97)	4.81 (4.48 to 5.17)		3.64 (3.57 to 3.72)
Immediate referral for colposcopy	1.14 (1.09 to 1.19)	1.08 (1.03 to 1.12)	1.11 (1.00 to 1.23)		1.10 (1.07 to 1.13)
Colposcopies	1.79 (1.72 to 1.85)	1.64 (1.58 to 1.70)	1.89 (1.75 to 2.05)		1.69 (1.65 to 1.73)
CIN2+	1.53 (1.45 to 1.62)	1.41 (1.33 to 1.51)	1.57 (1.32 to 1.87)		1.49 (1.43 to 1.55)
CIN3+	1.46 (1.36 to 1.56)	1.38 (1.28 to 1.50)	1.44 (1.14 to 1.82)		1.44 (1.36 to 1.51)
**Adjusted odds ratio for hrHPV *v *LBC (95% CI)**					
Positive screening test	3.60 (3.48 to 3.73)	4.02 (3.90 to 4.16)	5.12 (4.76 to 5.51)		3.90 (3.81 to 3.98)
Immediate referral for colposcopy	1.13 (1.08 to 1.18)	1.13 (1.09 to 1.18)	1.18 (1.07 to 1.31)		1.14 (1.11 to 1.17)
Colposcopies	1.77 (1.70 to 1.84)	1.73 (1.67 to 1.79)	2.07 (1.91 to 2.25)		1.77 (1.73 to 1.82)
CIN2+	1.50 (1.42 to 1.59)	1.45 (1.36 to 1.55)	1.67 (1.40 to 2.00)		1.49 (1.43 to 1.55)
CIN3+	1.44 (1.35 to 1.55)	1.42 (1.31 to 1.54)	1.54 (1.22 to 1.96)		1.44 (1.36 to 1.51)

See supplementary figure 1A for hrHPV testing and 1B for LBC details.

CIN2+=cervical intraepithelial neoplasia grade 2 or worse; CIN3+=cervical intraepithelial neoplasia grade 3 or worse

**Table 3:** Screening outcomes by the infecting human papillomavirus (HPV) genotype in three laboratories that managed women according to genotype at 12 month early recall

Outcome	HPV 16/18			Other HPV			Odds rato for HPV 16/18 *v* other HPV (95% CI)	
	No (%)	Total		No (%)	Total		Unadjusted	Adjusted
**No at baseline**								
Screened	5207 (4)	127 238		10 890 (9)	127 238		NA	NA
Positive cytology	2293 (44)	5207		2994 (27)	10 890		2.08 (1.94 to 2.22)	2.02 (1.89 to 2.17)
**Persistence of hrHPV infection**								
Negative cytology at baseline, persistence at 12 months	1639 (68)	2398		3624 (55)	6566		1.75 (1.58 to 1.93)	1.72 (1.56 to 1.90)*
Negative cytology at 12 months, persistence at 24 months	NA	NA		1368 (65)	2091		NA	NA
**Colposcopy outcomes**								
PPV for CIN2+ at baseline, positive cytology	1295 (57)	2254		840 (29)	2909		3.33 (2.96 to 3.73)	3.12 (2.78 to 3.52)
PPV for CIN2+ at 12 months, positive cytology	252 (48)	528		221 (27)	825		2.50 (1.98 to 3.14)	2.28 (1.80 to 2.89)
PPV for CIN2+ at 12 months, negative cytology	103 (13)	789		NA	NA		NA	NA
PPV for CIN2+ at 24 months, persistent hrHPV+	NA	NA		117 (10)	1144		NA	NA

See supplementary figure 1A for hrHPV testing details.

NA=not applicable; hrHPV=high risk human papillomavirus; PPV=positive predictive value; CIN2+= cervical intraepithelial neoplasia grade 2 or worse.

^*^ For clearance (=1-persistence), the unadjusted odds ratio was 0.57 (95% CI 0.52 to 0.63), and the adjusted odds ratio was 0.58 (95% CI 0.53 to 0.64).

Figure 1B shows that, among women screened with cytology, 4.0% had low grade abnormal and 1.4% had high grade abnormal cytology. After hrHPV triage of low grade cytological abnormalities, 3.8% (table 1) of all screened women had abnormalities that required colposcopy referral: 9.6% aged 24-29, 3.0% aged 30-49, and 1.2% aged 50-64 (table 2). Table 2 shows that, by comparison, after hrHPV screening, 4.2% of women were referred for colposcopy immediately because of concurrent positive cytology; 10.8% aged 24-29, 3.2% aged 30-49, and 1.4% aged 50-64. Table 3 shows that, compared with women with other high risk infections, women with HPV 16/18 infections were twice as likely to have concurrent positive cytology (44% *v* 27%, adjusted odds ratio 2.02, 95% confidence interval 1.89 to 2.17). Among the primary hrHPV positives with negative cytology, 2.8% were referred after early recall at 12 and 24 months, bringing the total proportion of women referred per protocol to 7.0%. Table 4 shows that among women who were hrHPV positive and cytology negative and recommended for early recall at 12 months, 83% attended, and amongst women with a persistent hrHPV positive and cytology negative test result who were recommended for retesting at 24 months, 76% so far attended. Attendance at colposcopy after immediate referral was 98% after screening with hrHPV and 94% after screening with cytology. It remained high after early recall at both 12 and 24 months, 96% and 89%, respectively.

**Table 4:** Compliance with per protocol referrals for colposcopy and early recall, and the associated positive predictive values (PPV) for cervical intraepithelial neoplasia grade 2 or worse (CIN2+)

Referral	hrHPV testing				Liquid based cytology		
	No referred	No attended (%)	CIN2+ (PPV)		No referred	No attended (%)	CIN2+ (PPV)
Immediate referral for colposcopy	7724	7542 (98)	3060 (41)^*^		15 117	14 250 (94)	High grade cytology: 4365 (86)^†^ Low grade abnormal: 1567 (17)^‡^ Combined: 5932 (42)
Early recall at 12 months	15 425	12 830 (83)	NA		NA	NA	NA
Colposcopy after early recall at 12 months	2914	2786 (96)	Negative cytology: 108 (13)^§^ Positive cytology: 690 (36)^¶^		NA	NA	NA
Early recall at 24 months	4399	3326 (76)	NA		NA	NA	NA
Colposcopy after early recall at 24 months	2156	1912 (89)	261 (14)		NA	NA	NA

hrHPV=high risk human papillomavirus; NA=not applicable.

See supplementary figure 1A for hrHPV testing and 1B for LBC details.

* The PPV was 87% (2115/2438) for women with concurrent high grade cytology and 19% (945/5104) for women with concurrent low grade cytology.

† PPV calculated for 5084 women with high grade cytology who attended colposcopy.

‡ PPV calculated for 9166 women with low grade cytology and a positive hrHPV triage test result who attended colposcopy.

§ PPV calculated for 849 women who were hrHPV positive with negative cytology at 12 month early recall who attended colposcopy.

¶ Positive cytology: low grade abnormalities or worse. PPV calculated for 1937 women with hrHPV who had positive cytology at 12 month early recall who attended colposcopy.

Table 3 shows that by the 12 month early recall, women with normal index cytology were less likely to become hrHPV negative if they were infected with HPV 16/18 compared with other genotypes ( 32% *v* 45%, adjusted odds ratio 0.58, 95% confidence interval 0.53 to 0.64). By 24 months, an additional 35% of women who were hrHPV positive with persistently negative cytology had cleared their infections.

Table 1 shows that cervical intraepithelial neoplasia grade 2 or worse was detected in 4156 (2.26%) women and cervical intraepithelial neoplasia grade 3 or worse in 2521 (1.37%) women screened with hrHPV testing. Approximately one in four of these lesions were detected after early recall of women who were HPV positive and cytology negative. Of the 101 women diagnosed with cervical cancer, 89 (88%) diagnoses were made after an immediate referral for colposcopy, suggesting that early recall after negative cytology was a safe strategy. Table 2 shows that detection of cervical intraepithelial neoplasia grade 2 or worse was strongly dependent on age, with the highest detection among women under 30 (6.6%), fourfold higher than at 30-49 (1.6%), and more than 10 times the detection at 50 or over (0.5%). Of all detected cervical intraepithelial neoplasia grade 2 or worse, 55% (2299/4156) were detected at 24-29, 39% (1638/4156) at 30-49, and 5% (219/4156) at 50-64 years of age. Among women screened with cytology, 3833 (1.0%) had cervical intraepithelial neoplasia grade 3 or worse. Cervical intraepithelial neoplasia grade 2 or worse was diagnosed in 6113 (1.5%) women, with 4.4% at 24-29, 1.1% at 30-49, and 0.3% at 50-64.

### Positive predictive value of referral to colposcopy

Table 4 shows that after immediate referral of women who were hrHPV positive and cytology positive, cervical intraepithelial neoplasia grade 2 or worse was detected in 41% (the positive predictive value was 19% for low grade and 87% for high grade cytology). After referral at 12 month early recall, cervical intraepithelial neoplasia grade 2 or worse was detected in 36% if cytology had become abnormal but only 13% if cytology remained negative with genotype 16/18 infection. At 24 month early recall, among all women who were persistently hrHPV positive, the positive predictive value of colposcopy establishing a diagnosis of cervical intraepithelial neoplasia grade 2 or worse was 14%. Among women screened with cytology, the positive predictive value was 86% for cervical intraepithelial neoplasia grade 2 or worse following high grade, and 17% following low grade cytology, giving a combined positive predictive value of 42%. On average, the positive predictive value for cervical intraepithelial neoplasia grade 2 or worse was 33% (4156/12 559) for colposcopies in hrHPV testing and 37% (6113/16 378) for colposcopies in liquid based cytology screening (adjusted odds ratio 0.78, 95% confidence interval 0.74 to 0.82; data not shown).

Table 3 shows that about 60% (1650/2828) of all detected cervical intraepithelial neoplasia grade 2 or worse were associated with HPV 16/18 infections (of which 94%, 1547/1650, of women had positive cytology either at baseline or at the 12 month early recall). After adjustment for key variables, the odds of a cervical intraepithelial neoplasia grade 2 or worse lesion diagnosed at colposcopy after abnormal index cytology were three times as high for HPV 16/18 infections than for other high risk genotypes (positive predictive value for cervical intraepithelial neoplasia grade 2 or worse 57% *v* 29%, adjusted odds ratio 3.12, 95% confidence interval 2.78 to 3.52). This difference remained at 12 month recall in women with persistent infections and incident positive cytology (48% *v* 27%, 2.28, 1.80 to 2.89). In women with negative cytology despite persistent HPV 16/18 infections, the positive predictive value of colposcopy at 12 months was far lower (13%) and among women with any persistent infection with other hrHPV genotypes at 24 months, the positive predictive value was 10%, and just 6% (data not shown) if cytology was negative.

### Comparison of hrHPV testing with cytology in the prevalence round

Table 1 shows that women screened with hrHPV testing were more likely to have a positive screening test that required further management (adjusted odds ratio 3.90, 95% confidence interval 3.81 to 3.98), and were also more likely to be referred for colposcopy immediately after screening (1.14, 1.11 to 1.17). The proportion of abnormal reflex cytology was 33% (7728/23 331, fig 1A). hrHPV testing led to a higher detection of cervical intraepithelial neoplasia grade 2 or worse at immediate referral (1.12 for cervical intraepithelial neoplasia grade 2 or worse, 1.07 to 1.17; 1.12 for cervical intraepithelial neoplasia grade 3 or worse, 1.06 to 1.19) and for cervical cancer (1.14, 0.88 to 1.48); this increase became significant with a larger number of screened women (adjusted odds ratio 1.21, 95% confidence interval 1.02 to 1.43; supplementary table 2).

Table 1 shows that when referrals after early recall at 12 and 24 months were included, the proportion of women with a colposcopy was significantly higher for hrHPV testing than for cytology (adjusted odds ratio 1.77, 95% confidence interval 1.73 to 1.82). The Mantel-Haenszel estimate controlling for all 240 strata was very similar, 1.79 (95% confidence interval 1.75 to 1.84). Also, the detection of cervical intraepithelial neoplasia was significantly higher with hrHPV testing. Compared with cytology, adjusted odds ratio for cervical intraepithelial neoplasia grade 2 or worse was 1.49 (95% confidence interval 1.43 to 1.55), for cervical intraepithelial neoplasia grade 3 or worse was 1.44 (1.36 to 1.51), and for cervical cancer 1.27 (0.99 to 1.63); also this difference reached statistical significance with a larger number of screened women, 1.32 (95% confidence interval 1.12 to 1.55; supplementary table 2). hrHPV testing did, however, involve more biopsies without an abnormality (adjusted odds ratio 1.98, 95% confidence interval 1.91 to 2.05) or with at most cervical intraepithelial neoplasia grade 1 (1.71, 1.62 to 1.82). The Mantel-Haenszel estimate for the detection of cervical intraepithelial neoplasia grade 2 or worse was consistent with the primary analysis, with an odds ratio controlling for 240 strata of 1.50 (95% confidence interval 1.44 to 1.57).

### Incidence screening round

By the end of 2014, 354 715 women aged 24-46 had been referred back to routine three year recall. To date, 122 032 (34%) have attended for a new primary screening test; 33% of the women were screened with hrHPV testing and 35% screened with cytology in the prevalence round (odds ratio for attendance adjusted for key variables 0.86, 95% confidence interval 0.85 to 0.87). Attendance at the incidence round was slightly higher in less deprived areas (adjusted odds ratio per decile of the index of multiple deprivation 1.03, 95% confidence interval 1.03 to 1.03).

Table 5 shows the outcomes for 110 523 women who were rescreened with the same test in the incidence round at three years. Most women had the same screening test as in the prevalence round. Among the 33 506 women screened with hrHPV testing in both rounds, 2271 (6.8%) had a positive screening test, just over half that seen in the prevalence round, with 495 (1.5%) immediately referred for colposcopy. Among the 77 017 women screened with cytology in both screening rounds, these proportions were 2.5% and 2.4%, respectively. Detection of cervical intraepithelial neoplasia grade 2 or worse was substantially lower in women screened with hrHPV testing (0.2% *v* 0.7% for cervical intraepithelial neoplasia grade 2 or worse, adjusted odds ratio 0.29, 95% confidence interval 0.22 to 0.38; 0.1% *v* 0.5% for cervical intraepithelial neoplasia grade 3 or worse, adjusted odds ratio 0.14, 95% confidence interval 0.09 to 0.23).

**Table 5:** Screening outcomes in the incidence round, by combination of tests in the prevalence and incidence screening rounds, among women aged 24-46 who were referred to routine recall at three years in the prevalence round.

Outcome	Prevalence and incidence round (%)*			Odds ratio for hrHPV testing *v* LBC (95% CI)	
	hrHPV	LBC		Unadjusted	Adjusted^†^
Incident episodes:					
Total	33 506 (100.0)	77 017 (100.0)		NA	NA
Negative in the prevalence round	33 407 (99.7)	77 017 (100.0)		NA	NA
Incidence round outcomes:					
Positive screening test	2271 (6.8)	1910 (2.5)		2.86 (2.69 to 3.04)	3.00 (2.82 to 3.20)
Immediate referral for colposcopy	495 (1.5)	1878 (2.4)		0.60 (0.54 to 0.66)	0.63 (0.57 to 0.70)
Any colposcopy	373 (1.1)	1608 (2.1)		0.53 (0.47 to 0.59)	0.57 (0.51 to 0.64)
CIN2+	61 (0.2)	541 (0.7)		0.26 (0.29 to 0.34)	0.29 (0.22 to 0.38)
CIN3+	19 (0.1)	349 (0.5)		0.12 (0.08 to 0.20)	0.14 (0.09 to 0.23)
Cervical cancer	0	15 (<0.1)		NA	NA

hrHPV=high risk human papillomavirus; LBC=liquid based cytology; NA=not applicable; CIN2+= cervical intraepithelial neoplasia grade 2 or worse; CIN3+= cervical intraepithelial neoplasia grade 3 or worse.

* A further 2075 women screened with hrHPV testing in the prevalence round were screened with LBC in the incidence round, and 9434 women were screened with LBC in the prevalence round and with hrHPV testing in the incidence round.

† Adjusted for age, index of multiple deprivation decile, and laboratory.

## Discussion

This pilot confirmed the findings of randomised trials and showed increased sensitivity for primary screening with hrHPV.^1^ ^2^ ^3^ ^4^ ^5^ hrHPV compared with liquid based cytology detected 50% more cervical intraepithelial neoplasia grade 2 or worse, 40% more cervical intraepithelial neoplasia grade 3 or worse, and 30% more cervical cancer in the prevalence round. A quarter of cervical intraepithelial neoplasia grade 2 or worse was detected after early recall in women with negative cytology, which clearly shows the added sensitivity of hrHPV testing seen across the entire age range. This increased sensitivity is reflected in the remarkably low detection of cervical intraepithelial neoplasia grade 2 or worse among women who were hrHPV negative when rescreened at three years, being only 29% and 14% of that after a prevalence round negative liquid based cytology, for cervical intraepithelial neoplasia grade 2 or worse and cervical intraepithelial neoplasia grade 3 or worse, respectively.

Our data suggest that the lower incidence of cervical cancer after a normal screening test reported from the randomised trials will be realised in the pilot.^3^ In the trials, this incidence was decreased by 70% up to eight years after screening (relative detection rate for hrHPV compared with cytology 0.30, 95% confidence interval 0.15 to 0.60).^3^ At present, 2500 women are diagnosed with cervical cancer each year in England, with a quarter diagnosed after negative cytology.^13^ Screening with hrHPV testing would translate to 400-500 fewer cases, or an about 20% decrease in the overall incidence, once hrHPV screening is rolled out nationally.^14^ The lower incidence of cervical intraepithelial neoplasia grade 3 or worse and cervical cancer at present screening intervals would strongly support the safety of extending the intervals to at least five years without increasing the risk of potentially life threatening disease.^3^ ^6^ The English Cervical Screening Programme, started in 1988, has been responsible for a decrease of 30% in cervical cancer incidence, but since 2002 this has plateaued. We would expect a further decrease after implementation of primary hrHPV screening when combined with the NHS HPV vaccination programme, which began in 2008.

Cytology triage of women who were hrHPV positive resulted in 7% of those screened being referred for a colposcopy, 4% immediately and 3% after early recall. Referral was high in women under 30, with almost 17% having a colposcopy, compared with 5% of those aged 30-49 and 3% of those aged 50-64. The increased demand for a colposcopy for women who were hrHPV positive and cytology negative is likely temporary. Our preliminary data from the incidence round show a halving of hrHPV positive rates compared with the prevalence round, as well as a reduction in cytological abnormalities. The referral rate will decrease further when younger birth cohorts enter the NHS Cervical Screening Programme in 2020 because over 80% of them will have been vaccinated against HPV 16/18.^15^

hrHPV positive with negative cytology appeared to be a safe basis on which to defer repeated testing of women who were hrHPV positive.^16^ The additional cases of cervical cancer diagnosed after early recall of women who were hrHPV positive and cytology negative at 12 and 24 months also support the relative safety of the implemented triage strategy, since these women would have been deferred to a routine recall at 36 or 60 months had their primary screening test remained liquid based cytology.

The coverage of the Cervical Screening Programme has decreased,^17^ and a switch to hrHPV testing should not exacerbate this. The screening processes and outcomes were monitored intensely during the pilot. We did not identify any serious incidents regarding women’s concerns or the practicability of large scale implementation of hrHPV testing. Adherence to colposcopy referral and early recall were both strong at 95% and 80%, respectively, and this will be an important element for achieving high sensitivity and cost effectiveness of hrHPV screening.^18^ Furthermore, early data from the incomplete incidence screening round shows a similar uptake of hrHPV testing and cytology screening. Uptake overall, however, remains lower in socioeconomically more deprived areas. Although not yet offered, self sampling facilitated by hrHPV testing represents a potential strategy to increase uptake.^19^

### Strengths and weaknesses of this study

Two key strengths of this study are its large size and the early recall protocol for women with negative cytology. This is the largest report of using hrHPV testing in any organised national programme in the developed world.^20^ ^21^ ^22^ ^23^ ^24^ ^25^ ^26^ ^27^ ^28^ ^29^ The largest report from Italy included 130 000 women screened with hrHPV testing and other reports were based on substantially smaller numbers of women or were limited to specific age groups, or both. We were able to identify even infrequent events with more accuracy in this large pilot, including the strongest data yet reported on outcomes from the subsequent incidence screening round(s). This confirms a very low incidence of cervical intraepithelial neoplasia grade 2 or worse and cervical cancer after the first round of screening with hrHPV testing. hrHPV was more prevalent in women screened in the English pilot than in most other studies because we included women as young as 24-29. This reiterates the importance of country specific data in informing a roll-out of hrHPV testing. Our study, which was conducted under conditions representative of a routine screening setting across England, has shown high clearance rates in women with negative cytology which avoids unnecessary colposcopy. It also showed that early recall of these women was adhered to by 80%, showing feasibility of this approach, ultimately leading to an uplift in the detection of cervical intraepithelial neoplasia grade 2 or worse by approximately 50%. Despite expected real life issues such as non-participation in screening and protocol violation, hrHPV screening in England performed impressively, and as expected from the results of pivotal randomised trials.

A limitation of the pilot, for reasons of practicability, was that the selection of women for hrHPV screening was based predominantly on geographical area and not on individual randomisation. Age and deprivation differences between women undergoing hrHPV and cytology screening were relatively small, although statistically significant owing to large numbers. All comparisons of the two screening tests were adjusted for information that could be obtained from routine laboratory registrations, although some residual confounding probably remained. We were unable to determine the total number of colposcopies undertaken for investigation of screening abnormalities, although the data on the number of women with at least one colposcopy should be highly complete as they are used to identify women overdue for their recommended colposcopies. We were also unable to link with information on women’s full screening history and records of screening invitations, and screening coverage does differ between geographical areas.^30^ All data were supplied by the laboratories and women could not be tracked if they had moved to another screening provider. Although completeness of follow-up remained high, some underreporting of detected disease probably exists. All cases of cervical cancer in England are monitored through a formal audit,^31^ and any cancers diagnosed after a negative hrHPV test result will be identified through that process.

### Conclusion

This pilot undertaken under routine screening conditions has confirmed that primary hrHPV cervical screening is practicable on a large scale and confers approximately 40% greater sensitivity for cervical intraepithelial neoplasia grade 3 or worse and approximately 30% greater sensitivity for cervical cancer than primary liquid based cytology. This increased detection in a prevalence round was followed by a marked reduction in the incidence after three years, supporting an extension of the screening intervals.

**What is already known on this topic**

More than 15 years of research with randomised controlled trials have produced a strong evidence base supporting the superiority of high risk human papillomavirus (hrHPV) testing for detection of cervical intraepithelial neoplasia grade 2 or worse compared with the current standard of liquid based cytology
Several countries have updated their screening guidelines and are switching from primary liquid based cytology with hrHPV triage to primary hrHPV testing with liquid based cytology triage
NHS England and Public Health England are working toward a national roll-out by the end of 2019

**What this study adds**

The pilot confirmed that prior screening with hrHPV testing is associated with a much lower incidence of high grade cervical intraepithelial neoplasia compared with liquid based cytology
Cervical screening intervals can be safely extended
hrHPV testing for cervical screening is practicable in England

## Web Extra

Extra material supplied by the author

**Supplementary materials**: Supplementary materials and table 1 and 2

**Supplementary materials:** Supplementary figure 1A

**Supplementary materials:** Supplementary figure 1B

**Supplementary materials:** Supplementary figure 2A

**Supplementary materials:** Supplementary figure 2B

## Notes

Contributors: JR, KD, JT, KE, JS, TG, VF, XT, AS, MH, PT, TL, JuP, RS, SM, and HK designed the study and had clinical oversight. KE, JS, CE, TG, VF, XT, JaP, KH, TL, and DS acquired the data. MR, CM, and SM performed the analysis. MR and HK drafted the manuscript. All authors provided critical comment. MR and HK submitted the monograph. The members of the participating laboratories were responsible for retrieving the data and transferring it to Queen Mary University of London and can take full responsibility for the integrity and the accuracy of the data specific to their laboratory. MR and CM had full access to the collated data in the study and can take responsibility for the integrity of the collated data and the accuracy of the data analysis. All authors were provided with all statistical reports and tables and could exercise full access to the collated data at Queen Mary University of London. MR and HK are the guarantors. The corresponding author attests that all listed authors meet authorship criteria and that no others meeting the criteria have been omitted.

Ethical approval: Women participating in the HPV Primary screening pilots were invited to make an informed choice on participating in the cervical screening programme. A decision is made to accept or decline a screening test based on access to accurate and up-to-date information on the condition being screened for, the testing process and potential outcomes. Specific information was provided at the invitation stage allowing for personalised informed choice. There was further opportunity to reflect on what the test and its results might mean when they attended for screening with the clinician taking the sample. Regulation 5, Health Service Regulations 2002, Confidentiality Advisory Group Reference: 15/CAG/0207, was the legal basis to process the data.

Data sharing: No additional data are available.

The manuscript’s guarantors (MR and HK) affirm that the manuscript is an honest, accurate, and transparent account of the study being reported; that no important aspects of the study have been omitted; and that any discrepancies from the study as originally planned have been explained.
