# Supplementary material for: Primary cervical screening with high risk human papillomavirus testing: observational study
Source: BMJ. 2019 Feb 6;364:l240. doi: 10.1136/bmj.l240 (PMC6364146; doi:10.1136/bmj.l240)
Supplement: Supplementary file 1 — Supplementary materials: Supplementary materials and table 1 and 2 [file rebn044972.ww1.pdf]

Centre for Cancer Prevention, Wolfson Institute of Preventive Medicine
Queen Mary University of London

26 April 2018

Primary cervical screening with high-risk human papillomavirus testing: First results from the English pilot implementation

Matejka Rebolj, Janet Rimmer, Karin Denton, John Tidy, Christopher Mathews, Kay Ellis, John Smith, Chris Evans, Thomas Giles, Viki Frew, Xenia Tyler, Alexandra Sargent, Janet Parker, Miles Holbrook, Katherine Hunt, Penny Tidbury, Tanya Levine, David Smith, Julietta Patnick, Ruth Stubbs, Sue Moss, Henry Kitchener

SUPPLEMENTARY MATERIAL

Data management

The codes used by laboratory management systems were formatted to unify the dataset. Records were identified as HR-HPV or cytology primary by the laboratory and were flagged as such in the database.

Women with missing or incomplete NHS numbers were excluded, unless the NHS number could be populated from a linked colposcopy using the screening test ID number.

Registration of the reason for taking the sample was considered unreliable, and we specified an algorithm to determine the sequence of prevalence-round primary screening, follow-up and incidence-round primary screening tests. Women were followed from their first sample evaluated in a participating laboratory during the pilot period (prevalence round).

The colposcopy records with information on outcomes (mostly as histology diagnoses) and results of referral smear (corresponding to NHS KC65 submission) were used to determine whether the woman attended colposcopy.

Screening episodes were created from the screening tests. Each woman's first screening test was considered the index test. If the routinely recorded outcome was for 'routine recall', the episode was closed. If the recorded outcome was for repeat testing or colposcopy referral, the episode remained open and had the next available test added to the episode. If a test result was invalid then the outcome was not recorded but flagged and the episode remained open. At most four screening tests were inspected. Once all screening tests were linked, colposcopy records up to one year after the final screening test were added to the episode. Any screening tests or colposcopies within two years of the episode finish date when a colposcopy had been recorded were considered follow-up tests, e.g. as part of the routine test of cure algorithm. This check was repeated three times. Any test after a routine recall outcome was considered the index test of a subsequent episode, i.e. an incidence episode, unless there was an intervening colposcopy record.

The NHS Postcode Directory was used to look up the Lower Layer Super Output Area. This was used to link to English government indices of deprivation 2015 report, to identify Index of Multiple Deprivation (IMD) deciles.

Supplementary figures

Supplementary figure 1. Flow diagram for prevalence episodes that started by 31st December 2014 (N=578,547) including per-protocol follow-up and other follow-up. Colposcopy data were available until 31st May 2017. A) Women screened with HR-HPV testing. B) Women screened with cytology. White rectangles: per-protocol follow-up. Shaded rectangles: Other follow-up.

Supplementary figure 2. Flow diagram for prevalence episodes that started by 31st May 2017 (N=1,532,908) including per-protocol follow-up and other follow-up. Colposcopy data were available until 31st May 2017. A) Women screened with HR-HPV testing. B) Women screened with cytology. White rectangles: per-protocol follow-up. Shaded rectangles: Other follow-up.

Supplementary tables

Supplementary table 1. Screening in the pilot until 31st May 2017: characteristics of samples in the prevalence round for women 24-64 years of age.

	Cytology-based screening	HR-HPV-based screening	Total
	N (%)	N (%)	N (%)
**Total**	1,090,734 (71%)	442,174 (29%)	1,532,908 (100%)
**Age (in years)**			
24-29	212,104 (71%)	85,739 (29%)	297,843 (100%)
30-49	608,890 (72%)	241,198 (28%)	850,088 (100%)
50-64	269,740 (70%)	115,237 (30%)	384,977 (100%)
Mean (SD)	41.0 (11.0)	41.3 (11.2)	41.1 (11.1)
**Laboratory**			
A	130,555 (70%)	56,396 (30%)	186,951 (100%)
B	93,069 (67%)	46,142 (33%)	139,211 (100%)
C	171,733 (77%)	52,602 (23%)	224,335 (100%)
D	389,570 (71%)	155,295 (29%)	544,865 (100%)
E	138,723 (80%)	35,730 (20%)	174,453 (100%)
F	167,084 (64%)	96,009 (36%)	263,093 (100%)
**IMD decile**			
1 (most deprived)	172,868 (77%)	52,683 (23%)	225,551 (100%)
2	119,493 (77%)	35,829 (23%)	155,322 (100%)
3	126,407 (74%)	44,384 (26%)	170,791 (100%)
4	121,558 (74%)	41,795 (26%)	163,353 (100%)
5	109,605 (68%)	51,019 (32%)	160,624 (100%)
6	108,160 (67%)	52,768 (33%)	160,928 (100%)
7	99,262 (69%)	45,275 (31%)	144,537 (100%)
8	95,081 (69%)	42,072 (31%)	137,153 (100%)
9	81,252 (67%)	40,238 (33%)	121,490 (100%)
10 (least deprived)	57,048 (61%)	36,111 (39%)	93,159 (100%)
**Calendar year**			
2013	137,257 (71%)	55,197 (29%)	192,454 (100%)
2014	257,320 (67%)	128,773 (33%)	386,093 (100%)
2015	247,970 (66%)	128,153 (34%)	376,123 (100%)

2016	316,127 (76%)	100,019 (24%)	416,146 (100%)
2017	132,060 (81%)	30,032 (19%)	162,092 (100%)

Distribution of age (in years), IMD decile and laboratory differed statistically significantly between the two screening tests ($\chi^2 < 0.0001$).

Supplementary table 2. Screening in the pilot until 31st May 2017: comparison of populations and prevalence round screening outcomes for HR-HPV testing vs. LBC.

	**HR-HPV testing**	**LBC**	**Unadjusted OR for HR-HPV testing vs. LBC (95% CI)**	**Adjusted OR for HR-HPV testing vs. LBC (95% CI)[a]**
**Total**	442,174 (29%)	1,090,734 (71%)	NR	NR
**Age at screening (years)**				
24-29	85,739 (29%)	212,104 (71%)	NR	NR
30-49	241,198 (28%)	608,890 (72%)	NR	NR
50-64	115,237 (30%)	269,740 (70%)	NR	NR
**Deprivation at screening**				
IMD deciles 1-5 (most deprived)	225,710 (26%)	649,931 (74%)	NR	NR
IMD deciles 6-10 (least deprived)	216,464 (33%)	440,803 (67%)	NR	NR
**Procedures**				
Positive screening test outcomes requiring additional testing[b]	54,551 (12.3%)	40,043 (3.7%)	3.69 (3.64 to 3.74)	3.95 (3.89 to 4.00)
Immediate referrals[c]	18,193 (4.1%)	40,037 (3.7%)	1.13 (1.11 to 1.15)	1.17 (1.15 to 1.19)
Referrals after repeated testing[c]	NR	7543 (1.7%)	NR	NR
Colposcopies[d]	24,184 (5.5%)	38,517 (3.5%)	1.58 (1.55 to 1.61)	1.64 (1.61 to 1.67)
**Histological outcomes, after immediate referral[c]**				
CIN2+	7037 (1.59%)	14,767 (1.35%)	1.18 (1.15 to 1.21)	1.21 (1.18 to 1.25)
CIN3+	4436 (1.00%)	9485 (0.87%)	1.16 (1.11 to 1.20)	1.19 (1.15 to 1.24)
Cervical cancer	206 (<0.1%)	431 (<0.1%)	1.18 (1.00 to 1.39)	1.21 (1.02 to 1.43)
**All histological outcomes[e]**				
Normal biopsy	11,324 (2.56%)	16,811 (1.54%)	1.68 (1.64 to 1.72)	1.69 (1.65 to 1.73)
CIN1	4037 (0.91%)	6255 (0.57%)	1.60 (1.54 to 1.66)	1.72 (1.65 to 1.79)
CIN2+	8658 (1.96%)	14,995 (1.37%)	1.43 (1.40 to 1.47)	1.48 (1.44 to 1.52)
CIN3+	5303 (1.20%)	9580 (0.88%)	1.37 (1.32 to 1.42)	1.41 (1.37 to 1.46)
Cervical cancer	227 (0.05%)	435 (0.04%)	1.29 (1.10 to 1.51)	1.32 (1.12 to 1.55)

[a] Adjusted for age (in years), IMD decile, and laboratory.

[b] HR-HPV screening: HR-HPV positive with a known cytological outcome. Cytology screening: HR-HPV positive low-grade abnormal cytology or high-grade abnormal cytology regardless of the HR-HPV status.

[c] Per protocol. Referrals or lesions detected after immediate referral in women among e.g. HR-HPV negative or cytology negative primary screening tests were not included here.

[d] Counted as one per woman, including colposcopies conforming to the screening recommendations and colposcopies in women with screening test results for which the screening recommendations did not include a referral for colposcopy.
[e] Includes biopsies taken per protocol (colposcopy after immediate referral or after early recall at 12 and 24 months), and biopsies taken outside of the protocol (see Supplementary figure 2 for details).
