# Supplementary figures and images for: Primary cervical screening with high risk human papillomavirus testing: observational study
Source: BMJ. 2019 Feb 6;364:l240. doi: 10.1136/bmj.l240 (PMC6364146; doi:10.1136/bmj.l240)

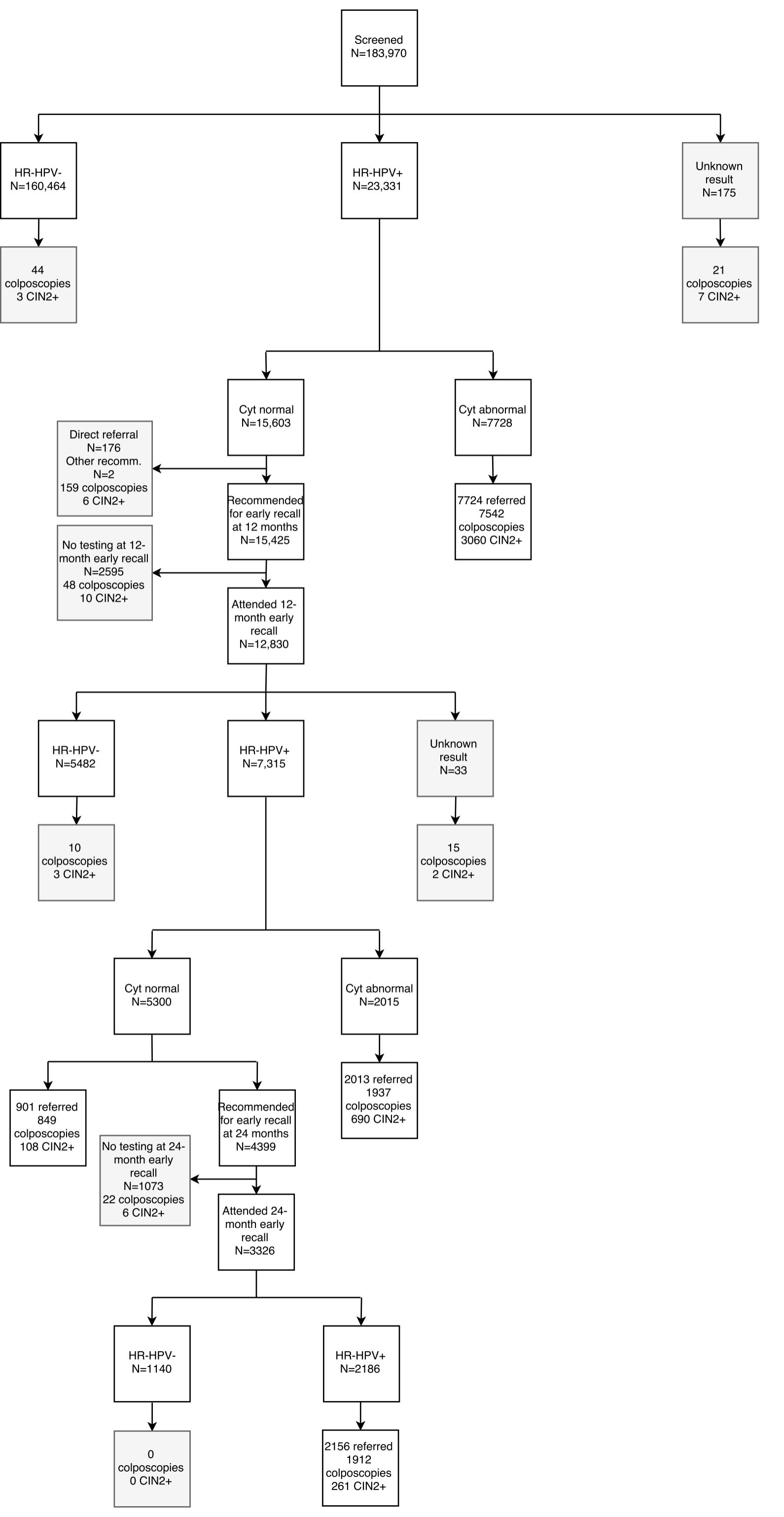

Supplement: Supplementary file 2 — Supplementary materials: Supplementary figure 1A [file rebn044972.ww2.pdf]

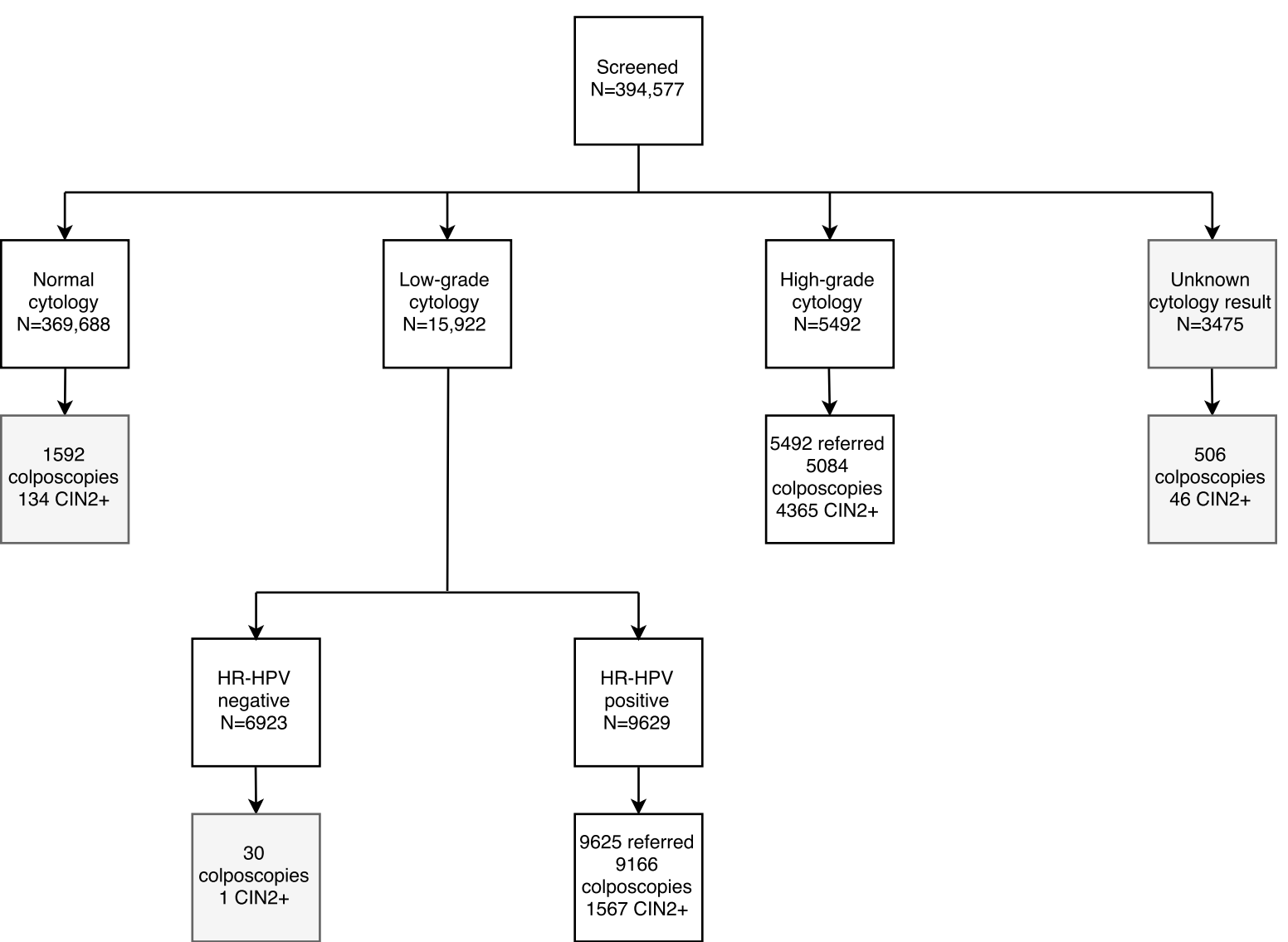

Supplement: Supplementary file 3 — Supplementary materials: Supplementary figure 1B [file rebn044972.ww3.pdf]

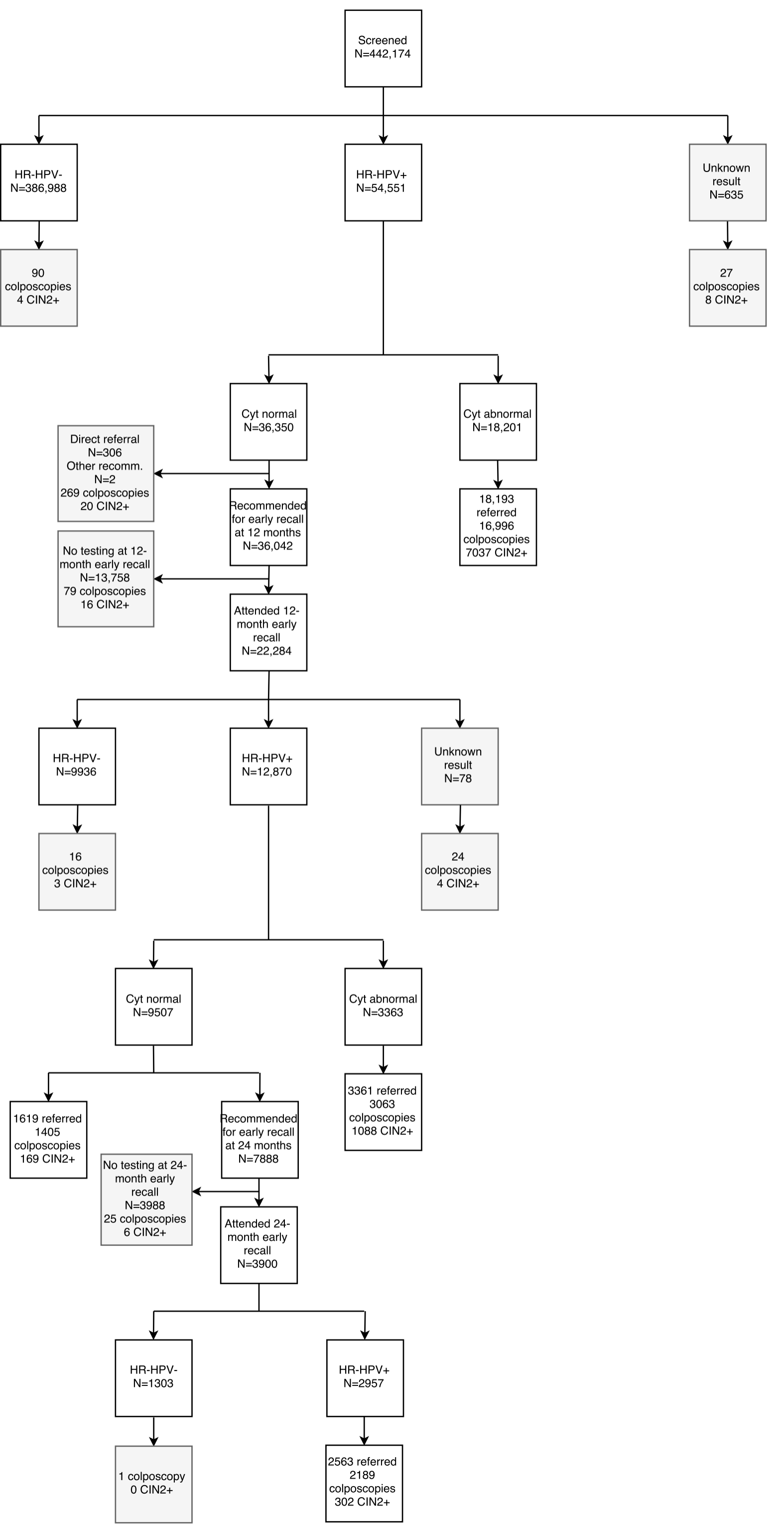

Supplement: Supplementary file 4 — Supplementary materials: Supplementary figure 2A [file rebn044972.ww4.pdf]

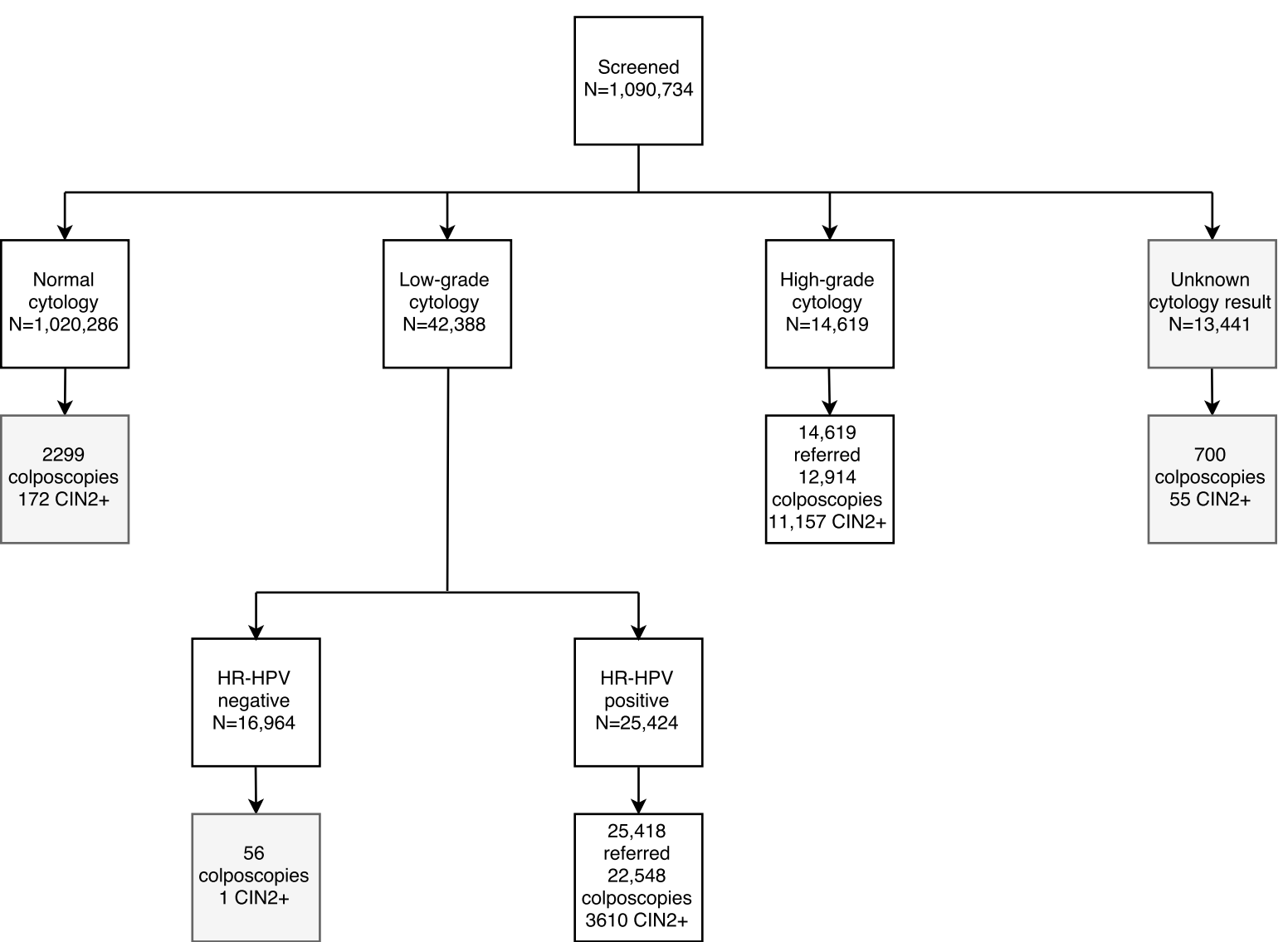

Supplement: Supplementary file 5 — Supplementary materials: Supplementary figure 2B [file rebn044972.ww5.pdf]
